# Activation of GPR39 with TC-G 1008 attenuates neuroinflammation via SIRT1/PGC-1α/Nrf2 pathway post-neonatal hypoxic–ischemic injury in rats

**DOI:** 10.1186/s12974-021-02289-7

**Published:** 2021-10-13

**Authors:** Shucai Xie, Xili Jiang, Desislava Met Doycheva, Hui Shi, Peng Jin, Ling Gao, Rui Liu, Jie Xiao, Xiao Hu, Jiping Tang, Lina Zhang, John H. Zhang

**Affiliations:** 1 Department of Critical Care Medicine, National Clinical Research Center for Geriatric Disorders, Xiangya Hospital, Central South University, Changsha, 410008 Hunan China; 2 Department of Physiology and Pharmacology, School of Medicine, Loma Linda University, Loma Linda, CA 92350 USA; 3 Department of Radiology, The Second People’s Hospital of Hunan Province/Brain Hospital of Hunan Province, Changsha, 410007 Hunan China; 4 Department of Neurosurgery, Chongqing Medical University, Yongchuan Hospital, Yongchuan, Chongqing, 402160 China; 5 Department of Intensive Care Unit, HuaShan Hospital, Fudan University, Shanghai, 200040 China; 6 Department of Neurosurgery, Affiliated Haikou Hospital, Xiangya School of Medicine, Central South University, Haikou, 570208 China; 7 Department of Neurology, Guizhou Provincial People’s Hospital, Guiyang, 550002 Guizhou China; 8 Department of Emergency, The Third Xiangya Hospital, Central South University, Changsha, 410013 Hunan China; 9 National Clinical Research Center for Geriatric Disorders, Xiangya Hospital, Central South University, Changsha, 410008 Hunan China; 10 Department of Neurosurgery and Anesthesiology, Loma Linda University Medical Center, Loma Linda, CA 92354 USA

**Keywords:** GPR39, TC-G 1008, Hypoxic–ischemic encephalopathy, Microglia, Neuroinflammation, SIRT1, PGC-1α, Nrf2

## Abstract

**Background:**

Hypoxic–ischemic encephalopathy (HIE) is a severe anoxic brain injury that leads to premature mortality or long-term disabilities in infants. Neuroinflammation is a vital contributor to the pathogenic cascade post-HIE and a mediator to secondary neuronal death. As a plasma membrane G-protein-coupled receptor, GPR39, exhibits anti-inflammatory activity in several diseases. This study aimed to explore the neuroprotective function of GPR39 through inhibition of inflammation post-hypoxic–ischemic (HI) injury and to elaborate the contribution of sirtuin 1(SIRT1)/peroxisome proliferator-activated receptor-γ coactivator 1α (PGC-1α)/nuclear factor, erythroid 2 like 2(Nrf2) in G-protein-coupled receptor 39 (GPR39)-mediated protection.

**Methods:**

A total of 206 10-day-old Sprague Dawley rat pups were subjected to HIE or sham surgery. TC-G 1008 was administered intranasally at 1 h, 25 h, 49 h, and 73 h post-HIE induction. SIRT1 inhibitor EX527, GPR39 CRISPR, and PGC-1α CRISPR were administered to elucidate the underlying mechanisms. Brain infarct area, short-term and long-term neurobehavioral tests, Nissl staining, western blot, and immunofluorescence staining were performed post-HIE.

**Results:**

The expression of GPR39 and pathway-related proteins, SIRT1, PGC-1α and Nrf2 were increased in a time-dependent manner, peaking at 24 h or 48-h post-HIE. Intranasal administration of TC-G 1008 reduced the percent infarcted area and improved short-term and long-term neurological deficits. Moreover, TC-G 1008 treatment significantly increased the expression of SIRT1, PGC-1α and Nrf2, but downregulated the expressions of IL-6, IL-1β, and TNF-α. GPR39 CRISPR EX527 and PGC-1α CRISPR abolished GPR39’s neuroprotective effects post-HIE.

**Conclusions:**

TC-G 1008 attenuated neuroinflammation in part via the SIRT1/PGC-1α/Nrf2 pathway in a neonatal rat model of HIE. TC-G 1008 may be a novel therapeutic target for treatment post-neonatal HIE injury.

**Supplementary Information:**

The online version contains supplementary material available at 10.1186/s12974-021-02289-7.

## Introduction

Hypoxic–ischemic encephalopathy (HIE) is a severe anoxic brain injury that leads to premature mortality or long-term disabilities in newborns, such as cerebral palsy, cognitive deficits, and mental retardation [1–3]. HIE contributed to 23% of neonatal deaths globally and has an incidence of 26 per 1000 live births in developing countries [4, 5]. The established standard of treatment for HIE is therapeutic hypothermia, but it can only provide limited neuroprotection [6]. In recent years, researchers have focused on finding new therapies to improve efficiency, enhance neuroprotection, and reduce side effects. For example, some peptides, activated by severe anoxic injury, might be involved in perinatal HIE’s pathophysiology and could be the potential therapeutic target for HIE.

As one of HIE pathophysiology’s main events, inflammatory responses take place within minutes post-HIE injury and mediate secondary neuronal death [7, 8]. The activation of neuroglial cells promotes the release of a large number of pro-inflammatory cytokines and reactive oxygen species (ROS), thereby leading to neuronal apoptosis. On the other hand, emerging evidence has demonstrated that inflammatory responses also play a beneficial role, and anti-inflammation is also one of the present neuroprotective agents’ main mechanisms. Activated microglia/macrophages can induce phagocytosis and the production of anti-inflammatory cytokines, which inhibits neuroinflammation and protects remaining viable neurons from death [9–12].

G-protein-coupled receptor 39 (GPR39), a plasma membrane G-protein-coupled receptor, was first cloned and identified in 1997 [13]. GPR39 is expressed in the gastrointestinal tract, amygdala, hippocampus, and auditory cortex, and zinc was thought to be a natural ligand of GPR39 [14, 15]. Activation of GPR39 and related subsequent signaling cascade has been identified in several cells and shown to regulate a vast array of physiological functions, such as proliferation, differentiation, ion transport and tight junction formation [16]. Moreover, activation of GPR39 has been demonstrated to promote wound healing, ameliorate symptoms of inflammatory bowel diseases, dampen epileptic seizure activity, reduce anxiety-like behaviors and regulate insulin secretion and malignant progression of several cancers [17–24]. Recently, accumulating in vitro evidence demonstrated that GPR39 exhibits anti-inflammatory activity by reducing the expression of pro-inflammatory cytokines (IL-1β, IL-6), enhancing anti-inflammatory cytokines production (IL-10), ameliorating oxidative stress and mitochondrial dysfunction [25–27].

A small amount of literature confirms that GPR39 might play a neuroprotective role in neuronal injury by inhibiting apoptosis and ameliorating endoplasmic reticulum stress [28–30]. However, whether GPR39 activation has protective and anti-inflammatory effects post-HIE remains unexplained. In the present study, we hypothesized that GPR39 agonist, TC-G 1008, attenuates inflammation via sirtuin 1(SIRT1)/PPARG coactivator 1 alpha (PGC-1α)/nuclear factor, erythroid 2 like 2(Nrf2), leading to improvement of neurological function in a rat model of neonatal HIE.

## Materials and methods

### Animals and model

In this study, all experimental protocols were approved by the Institutional Animal Care and Use Committee of Loma Linda University. All studies were performed in accordance with the United States Public Health Service’s Policy on Humane Care and Use of Laboratory Animals. Litters of Sprague Dawley rats, containing 12 pups and their mothers, were purchased from Envigo (Livermore, CA). A total of 206 10-day-old (P10) pups with body weights ranging from 16 to 23 g were used in this experimental study (Additional file 1: Fig. S1). The animal model used in this study is the Modified Rice Vannucci neonatal hypoxia–ischemia (HI) model [31]. Briefly, 3% isoflurane was used to anesthetize rat pups, and 2.5% isoflurane was used for maintenance during surgery. A small lateral incision (approximately 3–5 mm in length) was made to the right of the midline, across the sagittal plane. The upper and lower edges of the isolated right common carotid (CCA) artery were ligated with 5–0 silk thread and severed between the ligatures. All operations were completed within 10 min. However, for pups in the sham group we only isolated the right CCA. Pups were allowed to recover for an hour on a heated blanket, following which they were placed in an airtight jar partially immersed in a 37 °C constant temperature water bath, and exposed for 2.5 h to a gas mixture of 8% oxygen and 92% nitrogen. Pups in the sham group only got their CCA isolated without ligation and severance, and without undergoing hypoxia exposure.

### Experimental design

#### Experiment 1

To explore the time course expression levels of endogenous GPR39, SIRT1, PGC-1α and Nrf2 post-HIE, six time points (6 h, 12 h, 24 h, 48 h, 72 h, 7 days) were selected. The right (ipsilateral) brain samples were separate for protein extraction.

#### Experiment 2

To assess the neuroprotective effect of TC-G 1008 on pups post-HIE, three doses of TC-G 1008 were used. The groups included sham, HIE + vehicle, HIE + TC-G 1008(5 mg/kg), HIE + TC-G 1008 (15 mg/kg), HIE + TC-G 1008(45 mg/kg). TC-G 1008, suspended in 1% Tween in H_2_O, was administered intranasally at 1 h following HIE. Righting reflex, negative geotaxis tests and body weights were conducted at 48-h post-HIE. Rats were then killed and whole brain samples were separated for TTC staining or immunofluorescence staining.

#### Experiment 3

Long-term effects of TC-G 1008 treatment on neurological function was evaluated by neurobehavioral tests including foot-fault, rotarod test and water maze. Sham group, HIE + vehicle group and HIE + TC-G 1008 group (15 mg/kg) were included in the analysis. TC-G 1008 was administered at 1 h, 25 h, 49 h, and 73 h post-HIE. After the neurobehavioral tests were completed, rats were killed and whole brain samples were removed for Nissl staining.

#### Experiment 4

To analyze whether GPR39 receptor and signaling pathway-related proteins, SIRT1 and PGC-1α, participate in the underlying mechanism of TC-G 1008-mediated anti-neuroinflammation effects, CRISPR was used to inhibit GPR39 and PGC-1α, and EX527 was used to inhibit SIRT1. Rat pups were randomly divided into 8 groups, sham, HIE + vehicle, HIE + TC-G 1008 group, HIE + TC-G 1008 + control CRISPR, HIE + TC-G 1008 + GPR39 CRISPR, HIE + TC-G 1008 + PGC-1α CRISPR, HIE + TC-G 1008 + EX527. TC-G 1008 (optimal dose) or DDH_2_O were administered intranasally at 1 h following HIE induction. GPR39 CRISPR, control CRISPR or PGC-1α CRISPR (1 μg/pup) was given intracerebroventricularly at 48 h before HIE induction. EX527 (10 mg/kg) or DMSO was injected intraperitoneally at 1 h before HIE induction. Righting reflex, negative geotaxis tests, TTC staining, body weight, and immunofluorescence staining were conducted at 48-h post-HIE.

### Drug administration

TC-G 1008 (5, 15, 45 mg/kg, Tocris Bioscience, USA) was administered intranasally [32]. A total of 6 μl of TC-G 1008 or DDH_2_O was given every 2 min in alternating nares. EX527 (10 mg/kg, abcam, USA), or DMSO was injected intraperitoneally at 1 h before HIE. GPR39 CRISPR (Santa Cruz Biotechnology, USA), PGC-1α CRISPR (Santa Cruz Biotechnology, USA), or control CRISPR (Santa Cruz Biotechnology, USA) were given intracerebroventricularly (1.5 mm anterior, 1.5 mm lateral to the Bregma, and 1.7 mm deep on the ipsilateral hemisphere) at 48 h before HIE induction.

### Neurobehavioral tests

Following the principle of double-blind, neurobehavioral tests were performed by two investigators in an unbiased setup at either 48 h or 4 weeks post-HIE. Short-term behavioral tests include righting reflex and negative geotaxis were performed at 48-h post-HIE, while long-term behavioral tests rotarod, water maze, and foot-fault were performed at 4 weeks post-HIE.

In negative geotaxis test, pups were placed head downward on a 45-degree sloping board. The time from placing pups on the board to when pups rotated their bodies to head upward position was recorded. The maximum testing time was 1 min. In righting reflex test, the time from a back position that they were initially placed on to pups turning on all fours was recorded.

In foot-fault, rats were placed to walk on a horizontal grid floor (square size 20–40 cm with a mesh size of 4 cm^2^) elevated 1 m above ground for 1 min. The number of misplaced forelimbs or hindlimbs were recorded by video equipment (iponhe 6 s, USA).

In rotarod test, rats were placed on a rotating, horizontal, constant speed or accelerating rod (Columbus Instruments Rotamex, USA). The duration of rats on the rotarod was recorded by video equipment.

Morris water maze test was used to assess learning, memory, and visual ability on days 24–28 post-HIE. A hidden platform was set up by submerging it in a pool of water. Rats were trained to find the platform using visual cues around the room in both cued tests and hidden tests. If the rats could not reach the platform within 1 min, they would be manually guided to the platform. The probe experiment was scheduled on the 5th day after training and the platform was removed. The length of time it took the rats to reach the platform and the swimming distance of rats were tracked with the Video Tracking System SMART-2000 (San Diego Instruments Inc., USA). Subsequently, the distance, latency, and probe quadrant duration were quantified and analyzed.

### Infarct area measurements

Rat pups were anesthetized and euthanized at 48-h post-HIE. Brains were isolated, cut into 2-mm slices and stained with 2% solution of 2,3,5 triphenyltetrazolium chloride (TTC) (Sigma Aldrich, USA). Image J software (NIH) was used to analyze and calculate the infarct area. The percentage of infarct area = [(contralateral hemisphere − nonlesioned ipsilateral hemisphere)/2 × contralateral hemisphere] × 100% [33].

### Western blot

The expression levels of GPR39, SIRT1, PGC-1α and Nrf2 were measured at 0 h, 6 h, 12 h, 24 h, 48 h, 72 h and 7 days post-HIE by Western blot following the manufacturer’s recommendations [34, 35]. To analyze whether GPR39 receptor and SIRT1/PGC-1α/Nrf2 pathway were involved in the neuroprotective effects of TC-G 1008, the expression levels of GPR39, SIRT1, PGC-1α and Nrf2, and pivotal inflammatory cytokines IL-6, IL-1β, TNF-α were assessed via Western blot. RIPA lysis buffer (Santa Cruz Biotechnology, USA) was used to obtain whole cell lysates. Primary antibodies used were rabbit anti-GPR39 (1:500, Bioss), mouse anti-SIRT1 (1:2000, Abcam), rabbit anti-PGC-1α (1:1000, Abcam), rabbit anti-Nrf2 (1:1000, Abcam), rabbit anti-interleukin (IL)-1β (1:1000, Abcam), rabbit anti-interleukin (IL)-6 (1:1000, Abcam), mouse anti-TNF-α(1:500, Abcam) and mouse anti-β-actin(1:3000, Santa Cruz). The next day, the anti-rabbit (or anti-mouse) secondary antibodies (1:3000, Santa Cruz Biotechnology, USA) were incubated at room temperature for 1–2 h. The gray values were quantified and analyzed by Image J software (NIH).

### Histological analysis

Rats were deeply anesthetized and perfused with cold PBS solution followed by 4% formaldehyde solution through the heart at 48 h or 28 days post-HIE. The brains were isolated and post-fixed with 10% formalin (24 h), then transferred into 20% sucrose solution, followed by 30% sucrose solution for dehydration. The brains were sliced into 10-μm slices for immunofluorescence staining, or 20 μm for Nissl’s staining.

### Immunofluorescence staining

Immunofluorescence staining was conducted as described previously [36]. The 10-μm-thick brain slices were incubated with rabbit anti-GPR39 (1:50, Bioss), rabbit anti-interleukin (IL) -1β (1:100, Abcam), mouse anti-myeloperoxidase (MPO) (1:100, Abcam).The second day, the brain slices were incubated with the appropriate fluorescence-conjugated secondary antibodies (1:200) in the dark at room temperature. The stained sections were then visualized with a fluorescence microscope (Leica DMi8, Leica Microsystems, Germany), and photomicrographs of double-fluorescence labeling were merged to observe the expression of GPR39 on oligodendrocytes, and the staining positive cells of IL-1β and MPO were counted.

### Nissl staining

The 20-μm-thick brain slices were immersed in the following solutions, 95% ethanol for 2 min, 70% ethanol for 2 min, distilled water for 30 s, 0.5% cresyl violet (Sigma-Aldrich, USA) for 2 min, distilled water for 30 s, 100% ethanol and xylene for 1.5 min twice. Subsequently, the brain slices were mounted with DPX (Sigma-Aldrich, USA). The image of the brain slice was obtained by microscope (Olympus-BX51) equipped with MagnaFire SP 2.1B. Brain tissue loss was calculated with Image J software (NIH) and 3 brain slices in each brain were included in the analysis. The percentage of brain tissue loss = [(contralateral hemisphere − ipsilateral hemisphere)/contralateral hemisphere] × 100% [37, 38].

## Results

**Fig. 1:**
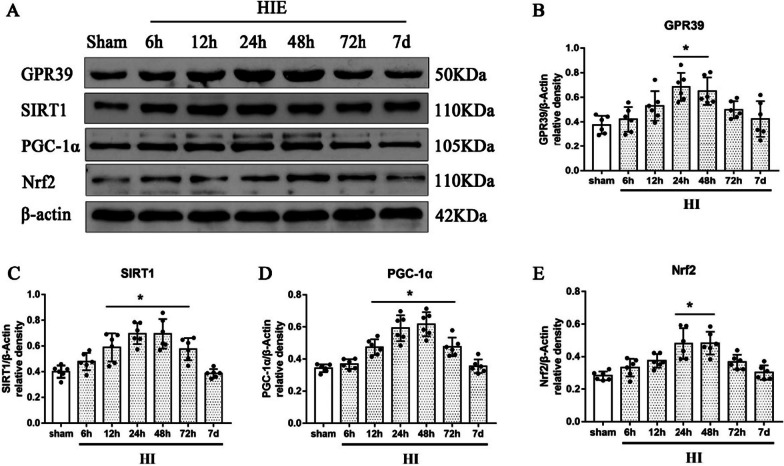
Temporal expression of GPR39/SIRT1/PGC-1α/Nrf2 in the brain post-HIE. **A** A representative western blot band of the time course. **B**-**E** The endogenous expression levels of GPR39, SIRT1, PGC-1α and Nrf2 were increased from 12 to 72 h, and peaked at 24 h or 48-h post-HIE. (Data are represented as mean ± SD; *p < 0.05 vs. sham, *n* = 6/group.)

**Fig. 2:**
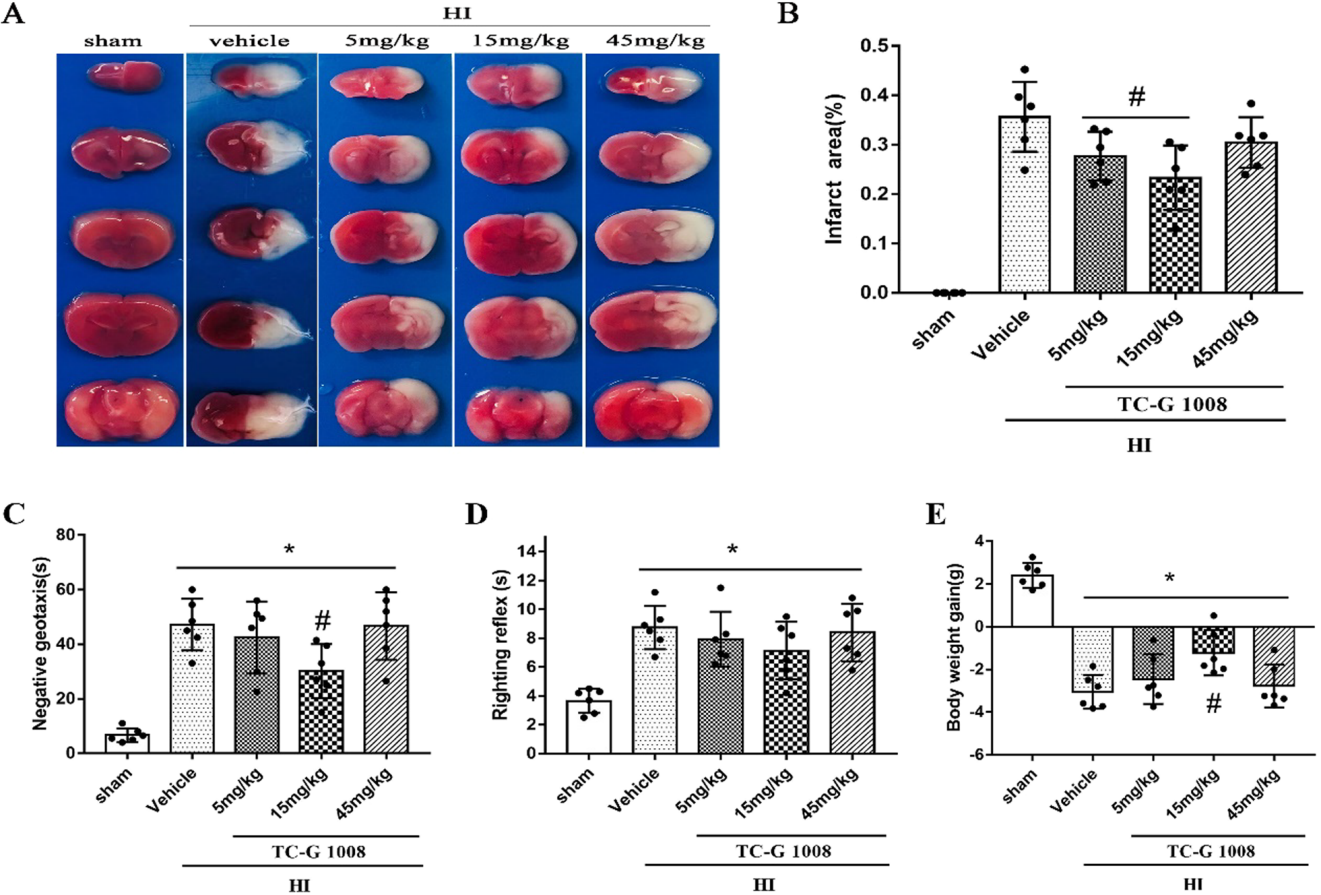
Effect of intranasal administration of TC-G 1008 on brain infarct are, short-term neurological function and body weight at 48-h post-HIE. (A-B) TTC staining showed that low (5 mg/kg) and medium (15 mg/kg) doses of TC-G 1008 significantly reduced infarct area when compared with vehicle. C–D Righting reflex and geotaxis reflex showed that middle dose (15 mg/kg) of TC-G 1008 significantly improved neurological function compared to vehicle animals. E TC-G 1008 significantly reduced HIE-induced body weight loss of rat pups. (Data are represented as mean ± SD; *p < 0.05 vs. sham; ^#^*p* < 0.05 vs. HIE + vehicle, *n* = 6/group)

**Fig. 3:**
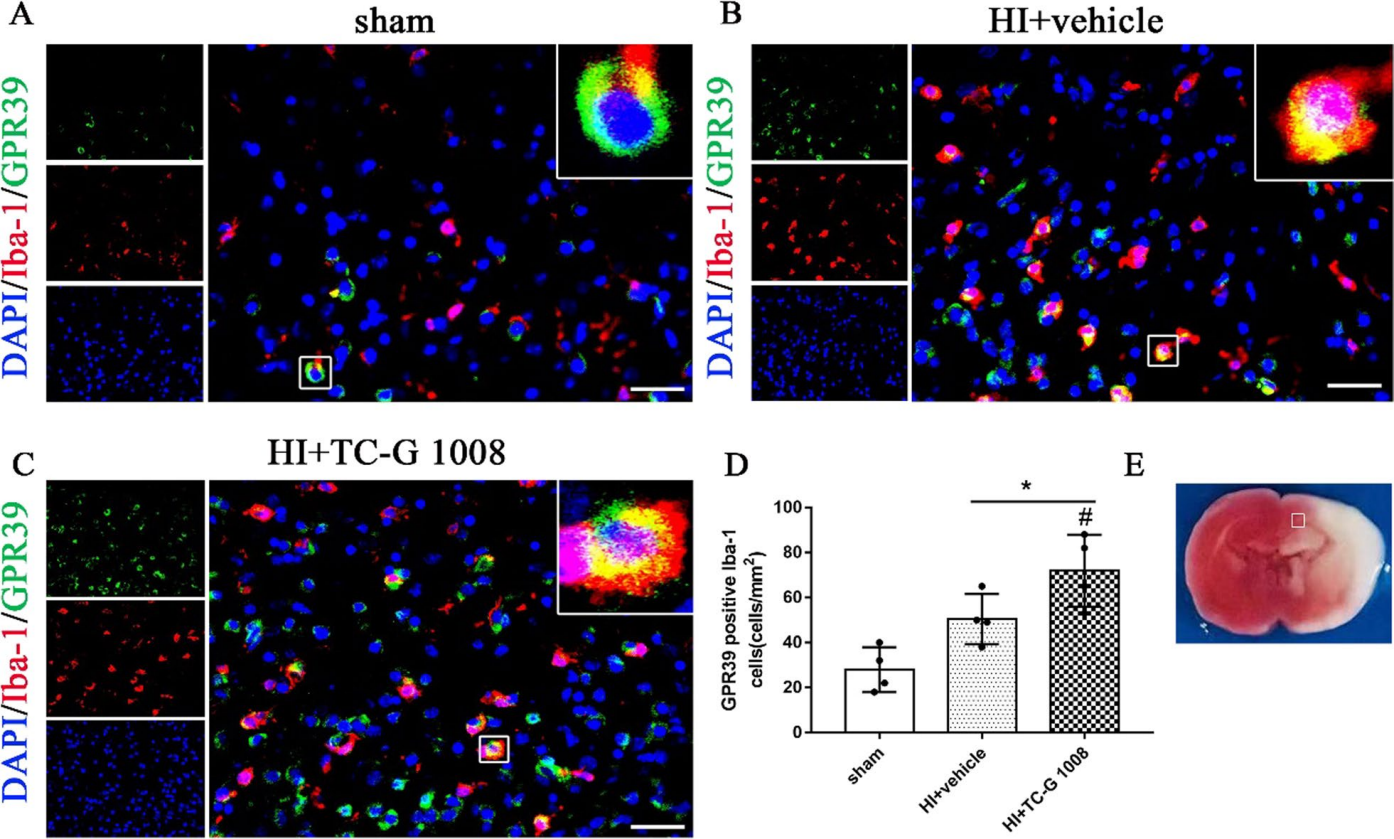
Representative immunofluorescence staining of GPR39 and the microglia marker Iba-1 in the brain at 48-h post-HIE. GPR39 was co-localized with Iba-1 in the sham (**A**), vehicle (**B**), and TC-G 1008 treatment (15 mg/kg) group (**C**), respectively. Compared with the sham group, GPR39 (green) expression was increased on microglia (red) in vehicle group and further increased post-TC-G 1008 treatment. Blue was for nucleus (DAPI). **D** Quantitative analysis of GPR39-positive Iba-1 cells in the ipsilateral cortex at 48-h post-HIE. E The schematic diagram shows the location of immunofluorescence staining (small white box). (Data are represented as mean ± SD; *p < 0.05 vs. sham; ^#^*p* < 0.05 vs. HIE + vehicle; *n* = 4/group; scale bar = 50 μm)

**Fig. 4:**
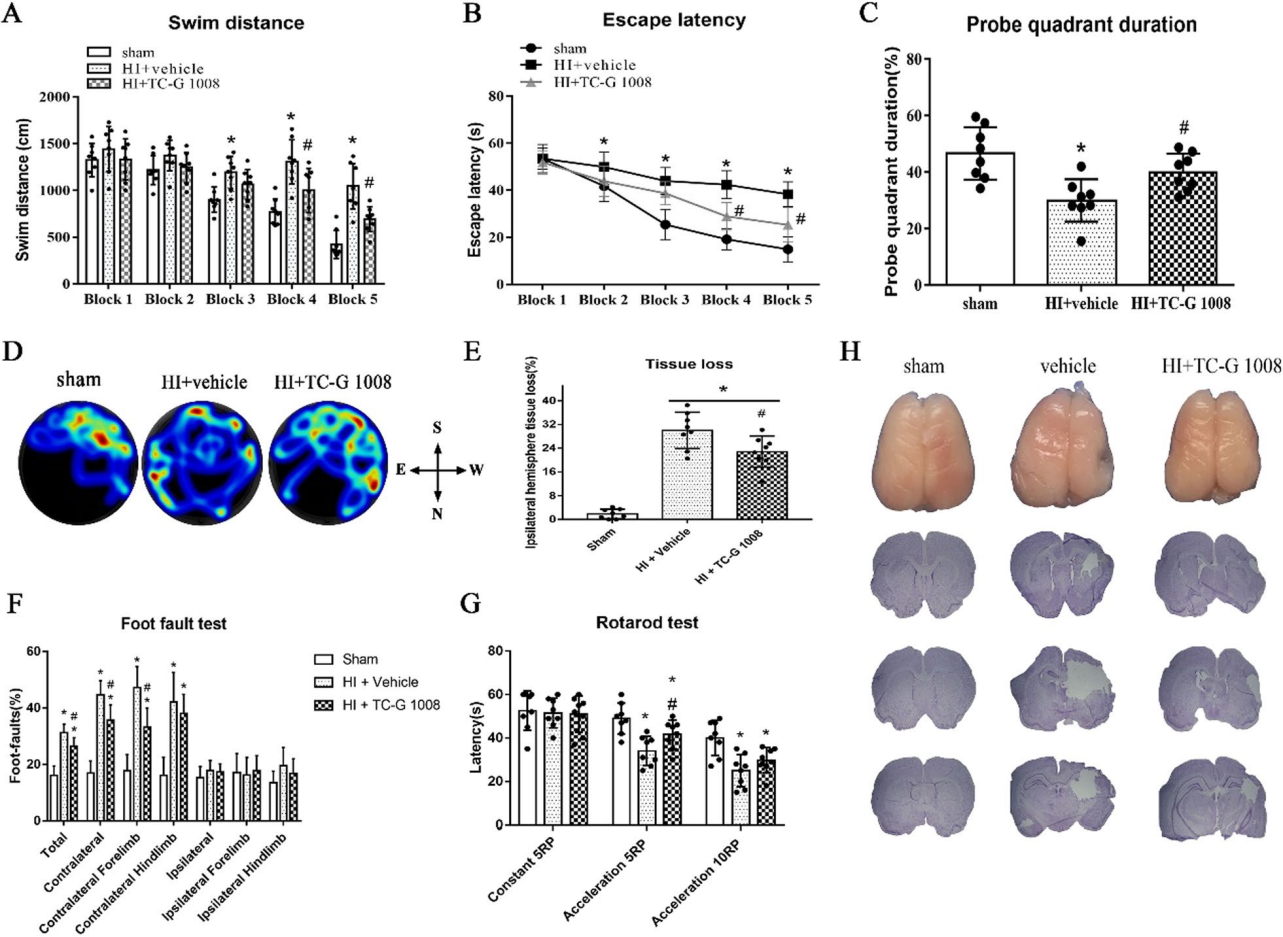
Effects of TC-G 1008 treatment on neurological function and brain atrophy at 4 weeks post-HIE. TC-G 1008 treatment group showed significant improvement in spatial memory loss as shown by shorter swimming distance (**A**), decrease in escape latency (**B**), and more time spent in the target quadrant during the probe trial (**C**, **D**).TC-G 1008 treatment significantly improved motor function as shown by foot-fault test (F) and rotarod test (**G**).TC-G 1008 treatment significantly reduced the percent of tissue loss (**E**, **H**) when compared with the vehicle

**Fig. 5:**
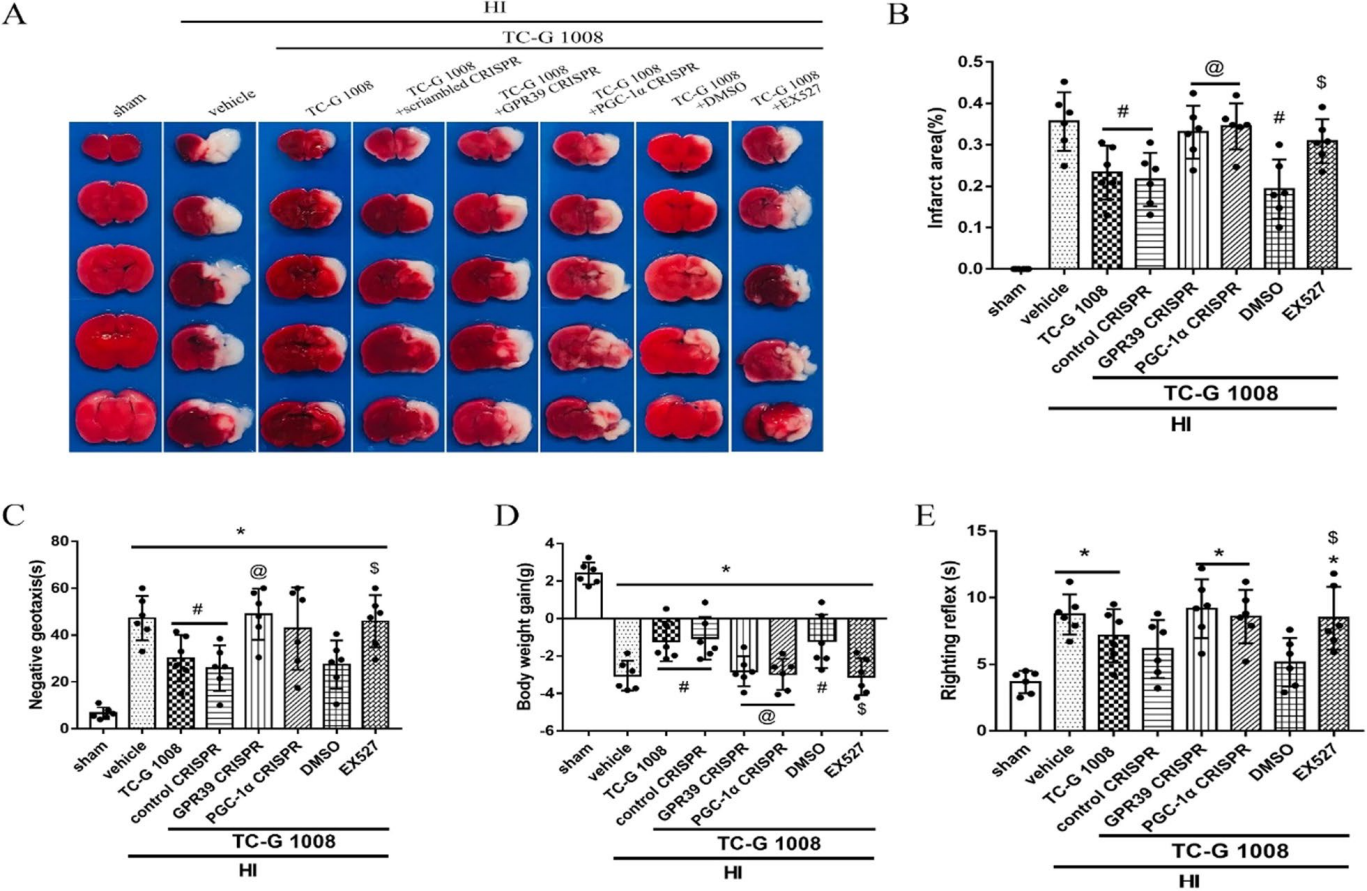
Effects of GPR39 and PGC-1α CRISPR, and SIRT1 inhibitor EX527 with TC-G 1008 on infarct volume, neurological function and body weight at 48-h post-HIE. **A**, **B** Animal groups treated with GPR39 CRISPR or PGC-1α CRISPR, or EX527 have significant greater proportion of infarction when compared with respective controls treated with TC-G 1008. **C**, **D** Animal groups treated with GPR39 CRISPR or PGC-1α CRISPR, or EX527 significantly abolished the neurological benefit of TC-G 1008 when compared with respective controls. **E** TC-G 1008 treatment or TC-G 1008 with DMSO significantly attenuated HIE-induced body weight loss, which were reversed by GPR39 CRISPR or PGC-1α CRISPR, or EX527. *p < 0.05 vs. sham; ^#^*p* < 0.05 vs. HIE + vehicle; ^@^*p* < 0.05 vs. HIE + TC-G1008 + control CRISPR; ^$^*p* < 0.05 vs. HIE + TC-G1008 + DMSO. *n* = 6 for each group

**Fig. 6:**
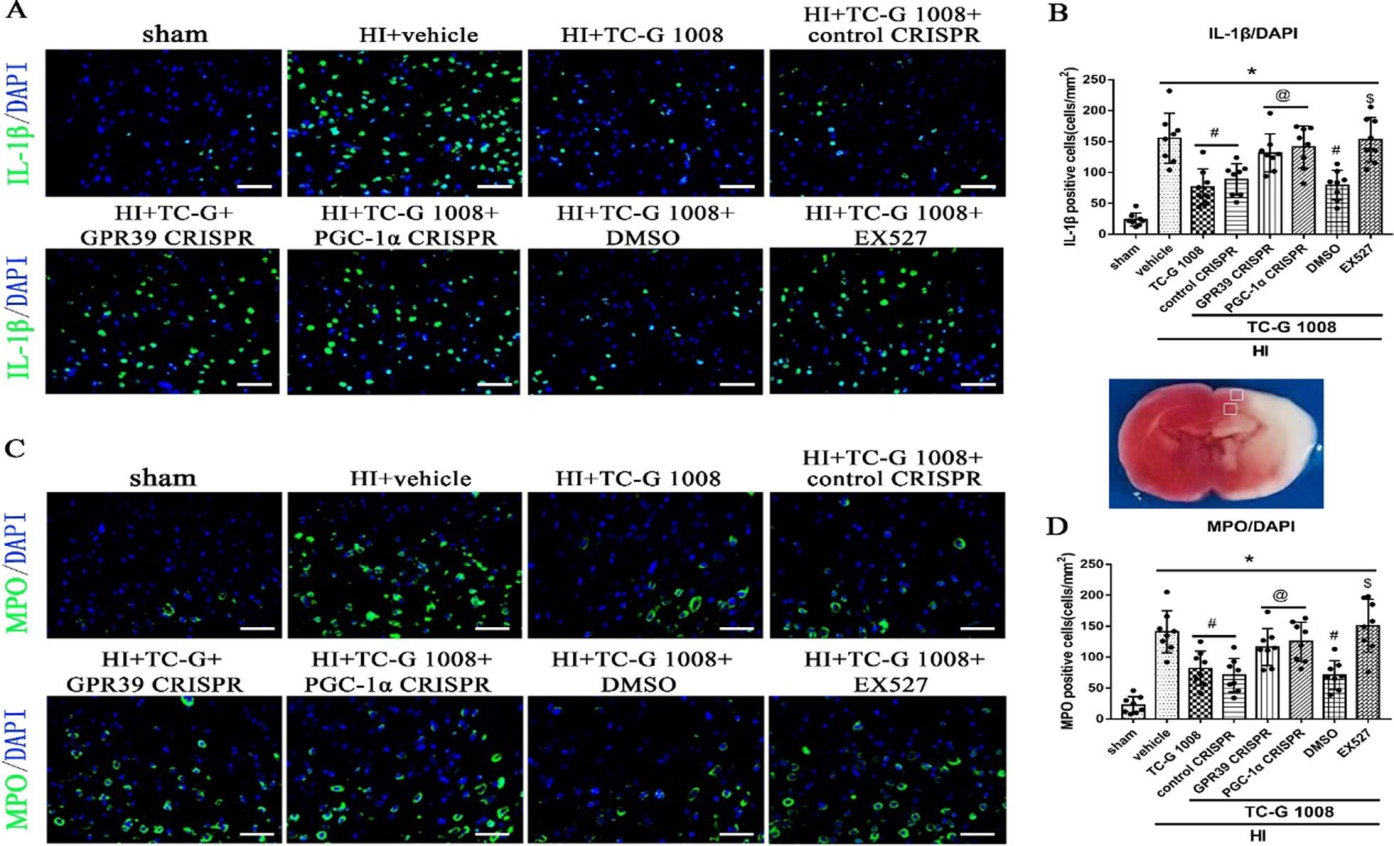
Effects of GPR39 and PGC-1α CRISPR, and SIRT1 inhibitor EX527 with TC-G 1008 on immunofluorescence staining of MPO and IL-1β at 48-h post-HIE. TC-G 1008 treatment or TC-G 1008 with DMSO significantly reduced the number of IL-1β/MPO-positive cells, which were abolished by either GPR39 CRISPR or PGC-1α CRISPR, or EX527. *p < 0.05 vs. sham; ^#^*p* < 0.05 vs. HIE + vehicle; ^@^*p* < 0.05 vs. HIE + TC-G1008 + control CRISPR; ^$^*p* < 0.05 vs. HIE + TC-G1008 + DMSO. *n* = 4 for each group

**Fig. 7:**
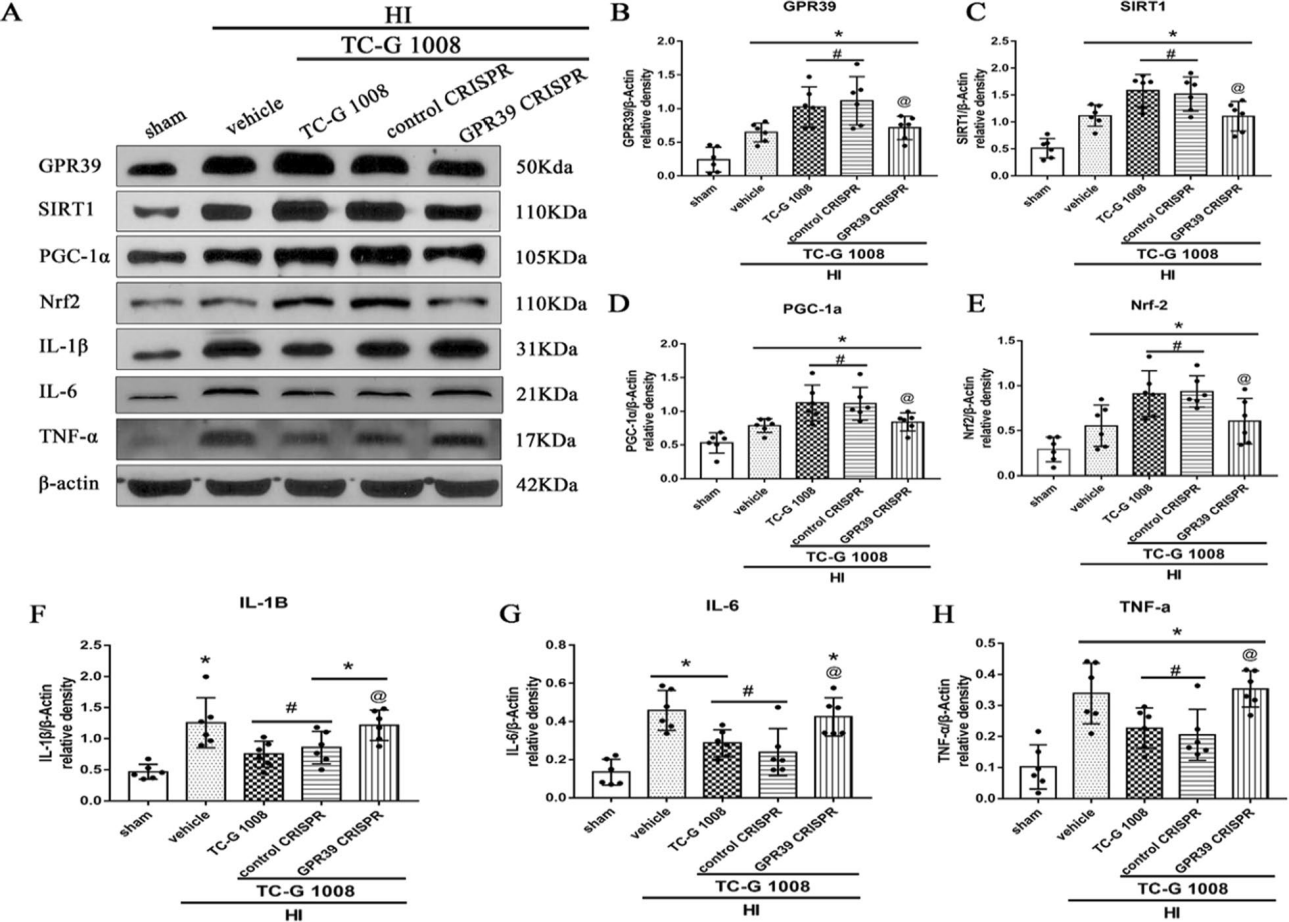
GPR39 CRISPR abolished anti-inflammatory effects of TC-G 1008 via GPR39/SIRT1/PGC-1α/Nrf2 signaling pathway at 48-h post-HIE. **A** Representative western blot bands. **B**–**H** Densitometric quantification of GPR39, SIRT1, PGC-1α, Nrf2, IL-1β, IL-6 and TNF-α in the ipsilateral hemisphere showed that TC-G 1008 treatment further upregulated the levels of GPR39, SIRT1, PGC-1α, and Nrf2, leading to less pro-inflammatory cytokines of IL-1β and TNF-α. GPR39 CRISPR significantly abolished such effects when administered with TC-G 1008. *p < 0.05 vs. sham; ^#^*p* < 0.05 vs. HIE + vehicle; ^@^*p* < 0.05 vs. HIE + TC-G1008 + control CRISPR, data are represented as mean ± SD, *n* = 6 for each group

1. Time course expression levels of endogenous GPR39, SIRT1, PGC-1α and Nrf2 post-HIE
  The endogenous expression levels of GPR39 receptor and pathway-related protein SIRT1, PGC-1α and Nrf2 were measured at six time points post-HIE. Compared with the sham group, GPR39, SIRT1, PGC-1α and Nrf2 were markedly increased from 12 to 72 h and peaked at 24 h or 48-h post-HIE (Fig. 1).
2. TC-G 1008 treatment reduced the percent infarcted area and improved short-term neurological function at 48-h post-HIE
  To investigate the neuroprotective effects of TC-G1008 treatment, three doses, low (5 mg/kg), medium (15 mg/kg), and high (45 mg/kg), were tested. From the TTC results, low and medium doses of TC-G 1008 showed to significantly reduce the percent infarcted area compared to vehicle group (Fig. 2A, B). The high-dose group did not show significant improvement compared to the vehicle group. In addition, medium dose of TC-G 1008 significantly reduced HIE-induced body weight loss of rat pups (Fig. 2E). Negative geotaxis test showed that medium dose of TC-G 1008 significantly relieved neurological deficits caused by hypoxia ischemia (Fig. 2C). However, same effect of TC-G 1008 treatment was not observed in the righting reflex (Fig. 2D). In our experiment, no unusual behavior was observed in pups post-administration of TC-G1008. Thus, these results implied that 15 mg/kg of TC-G1008 showed a more effective neuroprotective effect and was selected for further experiments.
3. Immunofluorescence staining showed the colocalization of GPR39 with microglia at 48-h post-HIE
  Double immunofluorescence staining of GPR39 receptor and Iba-1 (a marker for microglia) was carried out in the sham, vehicle and TC-G 1008 treatment group at 48-h post-HIE. As shown in Fig. 3, GPR39 was colocalized with microglia and the expression level of GPR39 on microglia was up-regulated in the vehicle group when compared with the sham group. Moreover, TC-G 1008 treatment further enhanced the expression of GPR39 on microglia.
4. TC-G 1008 improved long-term neurological function and reduced brain atrophy at 4 weeks post-HIE
  Foot-fault, rotarod and water maze tests were performed to evaluate the effects of TC-G 1008 treatment on the long-term neurological functions post-HIE.
  In the water maze test, compared with the control group, vehicle-treated animals were observed to spend more time and swim longer distances to find the platform, and less time in the platform quadrant. However, TC-G 1008 treatment relieved cognitive impairment, and improved memory and learning abilities compared with vehicle animals, as showed by the significantly shorter time it took for rats to find the platform (Fig. 4B), shorter swimming distance (Fig. 4A), and more time spent in the platform quadrant (Fig. 4C).
  Vehicle animals performed markedly worse compared with sham animals in foot-fault test, and TC-G 1008 treatment group showed to significantly reduce the total foot-faults and contralateral foot-faults when compared with the rats in the vehicle group (Fig. 4F). Furthermore, TC-G 1008 treatment significantly increased the falling latency at both 5 rpm and 10 rpm acceleration compared to vehicle in the rotarod test (Fig. 4G).
  The brain atrophy was evaluated by Nissl staining at 4 weeks post-HIE. The vehicle group displayed severe brain damage caused by HIE, characterized by brain tissue loss in ipsilateral hemisphere. It was significantly attenuated post-TC-G 1008 treatment when compared with vehicle group (Fig. 4E, H).
5. In vivo inhibition of GPR39, SIRT1 and PGC-1α abolished TC-G 1008’s neuroprotective effects at 48-h post-HIE
  To analyze whether GPR39 receptor and signaling pathway-related proteins, SIRT1 and PGC-1α, were involved in the neuroprotective effects of TC-G 1008, we inhibited GPR39 and PGC-1α using CRISPR, and SIRT1 with EX527.
  GPR39 CRISPR, PGC-1α CRISPR and EX527 reversed the protective effects of TC-G 1008, as shown from the significant increase in the percent infarcted area when compared to the corresponding control groups(Fig. 5A, B). Negative geotaxis and righting reflex tests showed that rat pups treated with TC-G 1008 in combination with either GPR39 CRISPR, PGC-1α CRISPR or SIRT1 inhibitor EX527 had markedly impaired neurological function compared with corresponding controls (Fig. 5C, D). Consistently, inhibition of GPR39, PGC-1α and SIRT1 abolished the effect of TC-G 1008 and significantly changed the weight of the animals when compared with corresponding treatment control groups (Fig. 5E).
  Moreover, animal groups treated with GPR39 CRISPR or PGC-1α CRISPR, or EX527 have significantly higher intensity levels of MPO and IL-1β than corresponding treatment control groups at 48 h post-HIE (Fig. 6).
6. In vivo inhibition of GPR39, SIRT1 and PGC-1α abolished the anti-neuroinflammatory effect of TC-G1008 through the GPR39/SIRT1/PGC-1α/Nrf2 signaling pathway at 48-h post-HIE
  Western blot data (Fig. 7A) showed that GPR39 receptor and pathway-related proteins SIRT1, PGC-1α, Nrf2, and pivotal inflammatory cytokines IL-6, IL-1β, TNF-α were up-regulated in vehicle group when compared with sham.TC-G 1008 treatment further increased the expression levels of GPR39, SIRT1, PGC-1α and Nrf2, but decreased the expression levels of IL-6,IL-1β and TNF-α when compared with vehicle group. Inhibition of GPR39 significantly decreased GPR39 expression, thereby abolishing the neuroprotective effects of TC-G 1008, resulting in a decrease in SIRT1, PGC-1α and Nrf2 expression, and an increase in IL-6, IL-1β and TNF-α expression.

In order to further explore the role of signaling pathway proteins in the anti-neuroinflammatory effect of TC-G 1008, SIRT1 inhibitor EX527 and PGC-1α CRISPR were used.

The results showed that EX527 abolished the effects of TC-G 1008, resulting in a decrease in PGC-1α and Nrf2 expression, and an increase in IL-6, IL-1β and TNF-α expression. Similarly, PGC-1α CRISPR significantly decreased PGC-1α expression, thereby abolishing the effects of TC-G 1008, resulting in a decrease in Nrf2 expression, and an increase in IL-6, IL-1β and TNF-α expression.

The trend of the western blot results of inflammatory factors was consistent with the findings of our immunofluorescence staining of IL-1β and MPO.

## Discussion

In this study, we aimed at evaluating the anti-inflammatory effects and the potential underlying mechanisms of GPR39 in a rat model of neonatal HIE. Our findings demonstrated that (1) the expression of GPR39 and pathway-related proteins, SIRT1, PGC-1α and Nrf2 were increased in a time-dependent manner, peaking at 24 h or 48-h post-HIE. (2) GPR39 was expressed in microglia at 48-h post-HIE. (3) Intranasal administration of TC-G 1008 (15 mg/kg) reduced the percent infarcted area and improved short-term and long-term neurological deficits. (4) TC-G 1008 attenuated neuroinflammation in part via the SIRT1/PGC-1α/Nrf2 pathway in a rat model of neonatal HIE.

In the central nervous system (CNS), it has been previously demonstrated that high GPR39 mRNA levels are present in the amygdala, hippocampus, and auditory cortex [15], with Zn^2+^ identified as the “physiological agonist” of GPR39 [39]. Extracellular Zn^2+^ activates ZnR/GPR39 receptor, thereby triggering multiple signaling pathways including, Gαs and Gαq/PLC/IP3 [40]. Previous studies indicated that a zinc-deficient diet led to the decreased expression of GPR39, and zinc supplementation for 4 weeks, significantly abolished the abnormal expression of GPR39 in the hippocampus [41]. Previous studies have reported that post-ischemia, neurons massively release extracellular Zn^2+^ to promote the production of pro-inflammatory cytokines [42]. In this study, we demonstrated that the expression of GPR39 was increased in a time-dependent manner post-HIE. Thus, the release of Zn^2+^ from neurons post-HIE may have led to the up-regulation of GPR39 expression that we observed.

As a specific agonist of GPR39, TC-G 1008 is widely used to explore the effect of GPR39 activation [25, 43, 44]. Previous studies have shown that treatment with TC-G 1008 (100 nM and 1 μM) enhanced keratinocyte proliferation through an ERK-dependent pathway [45]. Furthermore, TC-G 1008-mediated GPR39 activation promoted osteoblast differentiation and mineralization [43]. In a study where they investigated the effect of GPR39 on the intestinal barrier function, treatment with TC-G 1008 (1–10 μM) enhanced tight junction assembly in intestinal epithelial cells by PLC-CaMKKβ-AMPK pathways [46]. The expression of GPR39 in the hippocampus and hippocampal cells (HT-22) was upregulated following administration of TC-G 1008 [21, 28]. Advanced glycation end-products reduced GPR39 expression in a dose-dependent manner and TC-G 1008 reversed the effects of AGEs [47]. In this study, GPR39 was upregulated post-TC-G 1008 administration as seen from our immunofluorescence staining and mechanism studies, which is consistent with previous studies.

GPR39 plays an important role in wound healing, depression, inflammatory bowel diseases, alcohol use disorder, insulin secretion and several cancers [23, 46, 48–52]. However, the neuroprotective function of GPR39 has been partially confirmed. Studies have shown that GPR39 overexpression protected cells from undergoing cell death in a hippocampal cell line, and revealed its underlying mechanisms involving apoptosis and endoplasmic reticulum stress [29]. In another study, GPR39 exhibited its neuroprotective role by inhibiting apoptosis and thus protecting hippocampal neurons (HT-22) from corticosterone-induced injury [28]. Furthermore, it was observed that the zinc diet-treated group increased the expression of GPR39 and BMP protein, and improved cognitive impairment, while it showed to decrease hippocampal mossy fiber regenerative sprouting [41]. Therefore, GPR39 has been proposed as a potential therapeutic target for ischemia/reperfusion injury and neurodegenerative diseases [29]. In the present study, low and medium dose of TC-G 1008 showed to significantly reduce the percent infarcted area compared to vehicle group (Fig. 2A, B). In addition, medium dose of TC-G 1008 significantly reduced HIE-induced body weight loss of rat pups (Fig. 2E). As shown in rats’ performance in behavioral tests, TC-G 1008 improved long-term neurological function (Fig. 4). The behavioral tests were selected based on the neurological function that was being assessed. Negative geotaxis was used to evaluate reflex development, motor skills and vestibular labyrinth, and cerebellar integration, while righting reflex was used to reflect the muscle strength and subcortical maturation [53]. The rotarod test was used to assess the motor coordination of rodents and is especially sensitive in detecting cerebellar dysfunction [54]. The number of foot-faults indicates impaired movement correction and increased reaction time [55]. Morris water maze test was used to assess learning, memory, and visual ability. In general, activation of GPR39 with TC-G 1008 reduced the percent infarcted area and improved short-term and long-term neurological deficits.

Although the underlying mechanism is not completely understood, inflammation is one of the main contributors to the pathogenic cascade post-HIE. Hypoxia–ischemia initiates the inflammatory reaction in the brain parenchyma and the peripheral immune system, which mediates secondary brain injury and can last for a few days [7, 8]. Therefore, new agents that target inflammation will open new avenues for therapy of neonatal hypoxic–ischemic brain injury. GPR39, a recently discovered zinc-sensing receptor, has been proven to have anti-inflammatory effects in several studies [25–27, 56, 57].

**Fig. 8:**
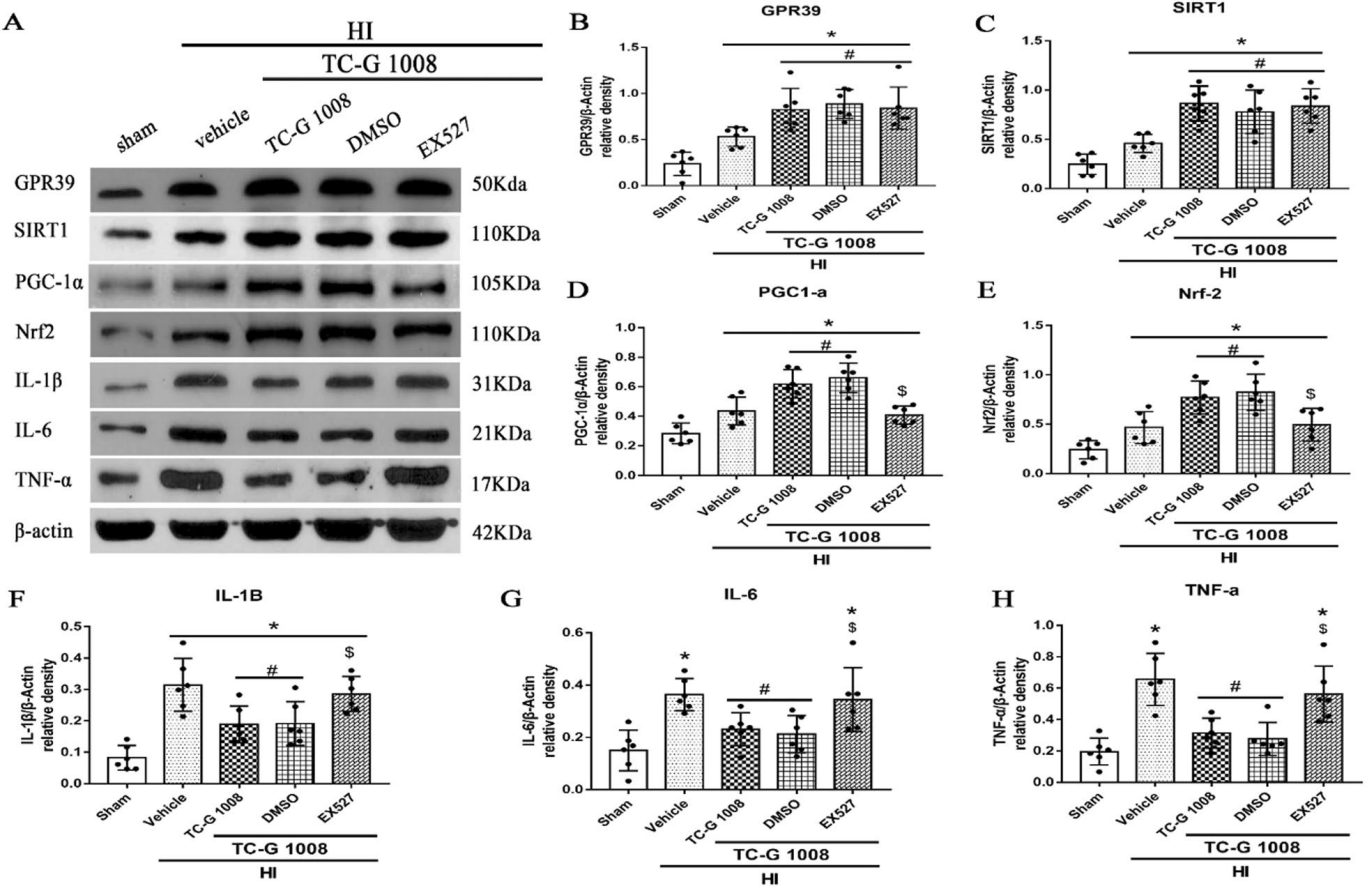
SIRT1 inhibitor EX527 abolished the anti-inflammatory effects of TC-G 1008 via GPR39/SIRT1/PGC-1α/Nrf2 signaling pathway at 48-h post-HIE. **A** Representative western blot bands. **B**–**H** Densitometric quantification of GPR39, SIRT1, PGC-1α, Nrf2, IL-1β, IL-6 and TNF-α in the ipsilateral hemisphere showed that EX527 significantly abolished the effects of TC-G 1008 treatment, resulting in a decrease in PGC-1α and Nrf2 expression, and an increase in IL-6, IL-1β and TNF-α expression.*p < 0.05 vs. sham; ^#^*p* < 0.05 vs. HIE + vehicle; ^@^*p* < 0.05 vs. HIE + TC-G1008 + control CRISPR, data are represented as mean ± SD, *n* = 6 for each group

**Fig. 9:**
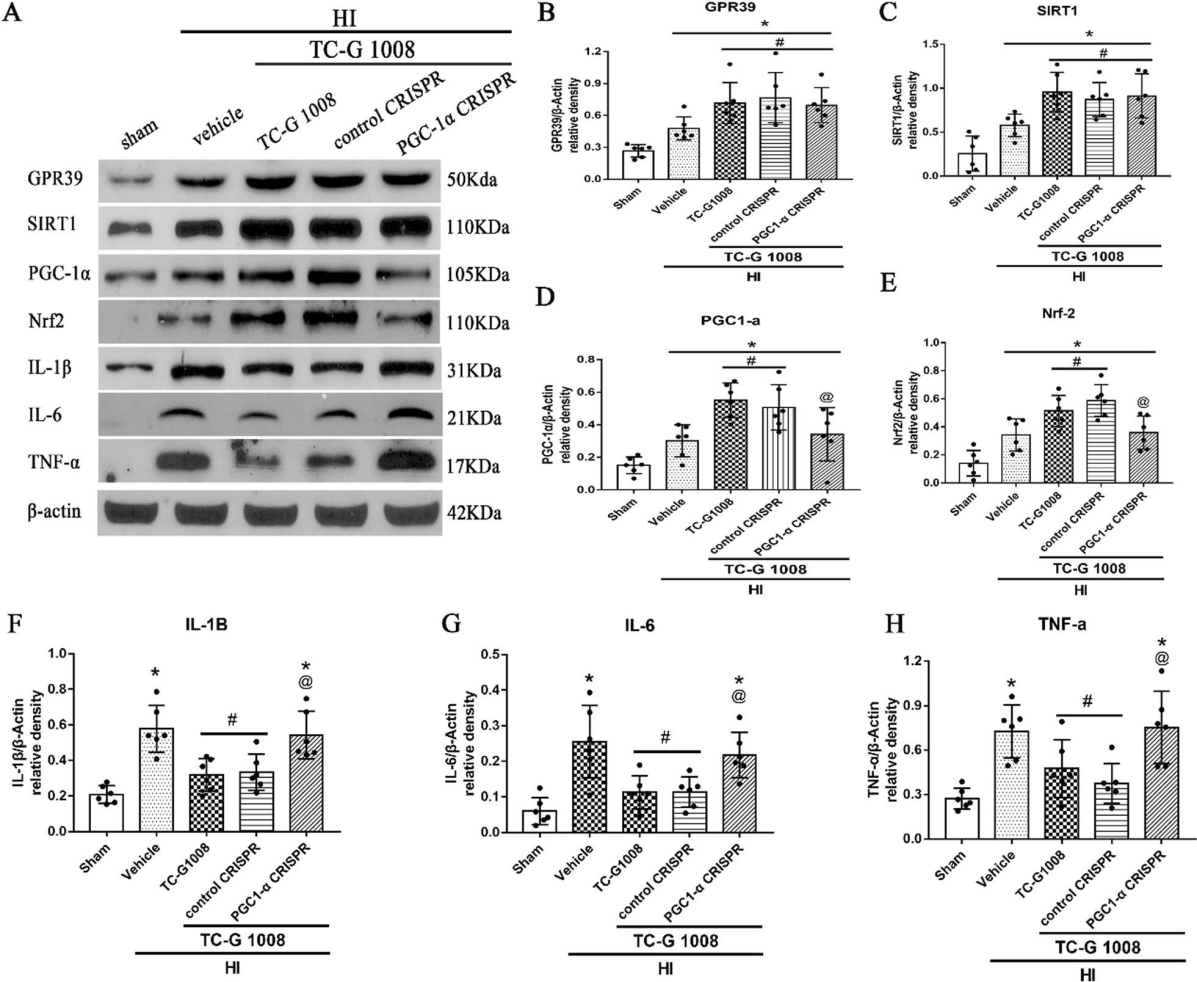
PGC-1α abolished the anti-inflammatory effects of TC-G 1008 via GPR39/SIRT1/PGC-1α/Nrf2 signaling pathway at 48-h post-HIE. **A** Representative western blot bands. **B**–**H** Densitometric quantification of GPR39, SIRT1, PGC-1α, Nrf2, IL-1β, IL-6 and TNF-α in the ipsilateral hemisphere showed that PGC-1α significantly abolished the effects of TC-G 1008 treatment, resulting in a decrease in Nrf2 expression, and an increase in IL-6, IL-1β and TNF-α expression.*p < 0.05 vs. sham; ^#^*p* < 0.05 vs. HIE + vehicle; ^$^*p* < 0.05 vs. HIE + TC-G1008 + DMSO, data are represented as mean ± SD, *n* = 6 for each group

GPR39 mediates synovial inflammation by ameliorating the expression of pro-inflammatory cytokines such as IL-βand IL-6 [25]. In the process of GPR39 regulating the activity of endothelial cells, Zn + is involved in the regulation of some inflammation-related key molecules including heme oxygenase-1, selectin L and IL-10 [56]. GPR39 was upregulated in thioglycollate-induced peritoneal macrophages and exerted its anti-inflammatory effects by increasing the production of IL-10 [27]. It has been shown that treatment with TC-G 1008 abolished the increased expression of pro-inflammatory cytokines induced by ox-LDL in the prevention of atherosclerosis [26]. However, in the process of promoting wound healing, the activation of GPR39 has the effect of promoting inflammation by increasing the production of pro-inflammatory cytokines IL-6 [57]. In the present study, we first discovered its anti-inflammatory effect in the brain by reducing the production of IL-6, IL-1β and TNF-α, as showed from the result of immunofluorescence staining of MPO and IL-1β and western blotting (Figs. 6, 7, 8, 9).

Numerous studies have shown that activation of SIRT1 is a neuroprotective agent for ischemic stroke through several mechanisms [58–61]. TC-G 1008 treatment mitigated IL-1b-induced inhibition of SIRT1, and the effect of TC-G 1008 on p53 acetylation and chondrocyte senescence were abrogated when SIRT1 was silenced [44]. Previous studies have indicated that PGC-1α was a major regulator of ROS metabolism and mitochondria biogenesis, which is closely related to the pathology of ischemic diseases and neurodegenerative diseases [62]. Several studies focusing on ischemic injury implied that the expression and activity of PGC1-α are at least partially dependent on SIRT1. Icariin has a neuroprotective effect in mice subjected to post-MCAO via increasing the SIRT1 and PGC-1a expression [63]. Ghrelin significantly attenuates brain damage post-HIE via the GHSR-1α/AMPK/Sirt1/PGC-1α/UCP2 signaling pathway [64]. Activation of the PGC-1α/Nrf-2/HO-1 signaling pathway plays a critical role in tannic acid (TA) administration against traumatic brain injury through reducing oxidative damage, mitochondrial impairment, and inflammation [65]. In addition, the expression levels of SIRT1 PGC1-a and Nrf2 were significantly up-regulated post-HIE [64, 66], which is consistent with the results observed in our study. The activation of GPR39 increased the expression of SIRT1, PGC-1α and Nrf2, and reduced the expression of pro-inflammatory cytokines (Fig. 7), but this effect was reversed by GPR39 CRISPR. EX527 and PGC-1α CRISPR abolished the effects of TC-G 1008, resulting in a decrease in PGC-1α and Nrf2 expression, and an increase in IL-6, IL-1β and TNF-α expression. Similar effects have also been observed in our immunofluorescence staining experiments of IL-1β and MPO. Therefore, the anti-inflammatory effect of GPR39 is partly dependent on the SIRT1/PGC-1α/Nrf2 signaling pathway post-HIE. The role of other neuroprotective functions and underlying mechanisms will need to be further investigated.

## Conclusions

In conclusion, intranasal administration of TC-G 1008 reduced the percent infarcted area and improved short-term and long-term neurological deficits post-HIE. TC-G 1008 attenuated neuroinflammation in part via the SIRT1/PGC-1α/Nrf2 pathway in a rat model of neonatal HIE. TC-G 1008 may be a novel therapeutic target for treatment post-neonatal HIE injury. Activating GPR39 may be a promising therapeutic target to attenuate neuroinflammation post-neonatal HIE.

## Supplementary Information

**Additional file 1:** Details of animals used in this study.

## Data Availability

The data supporting the findings of this study are available from the corresponding author upon reasonable request.

## Abbreviations

CCA: Common carotid
CNS: Central nervous system
GPR39: G-protein-coupled receptor 39
HIE: Hypoxic–ischemic encephalopathy
MPO: Myeloperoxidase
Nrf2: Nuclear factor, erythroid 2 like 2
PGC-1α: Peroxisome proliferator-activated receptor-γ coactivator 1α (PGC-1α)
ROS: Reactive oxygen species
SIRT1: Sirtuin 1
TA: Tannic acid
