# Supplementary figures and images for: Activation of GPR39 with TC-G 1008 attenuates neuroinflammation via SIRT1/PGC-1α/Nrf2 pathway post-neonatal hypoxic–ischemic injury in rats
Source: J Neuroinflammation. 2021 Oct 13;18:226. doi: 10.1186/s12974-021-02289-7 (PMC8513331; doi:10.1186/s12974-021-02289-7)

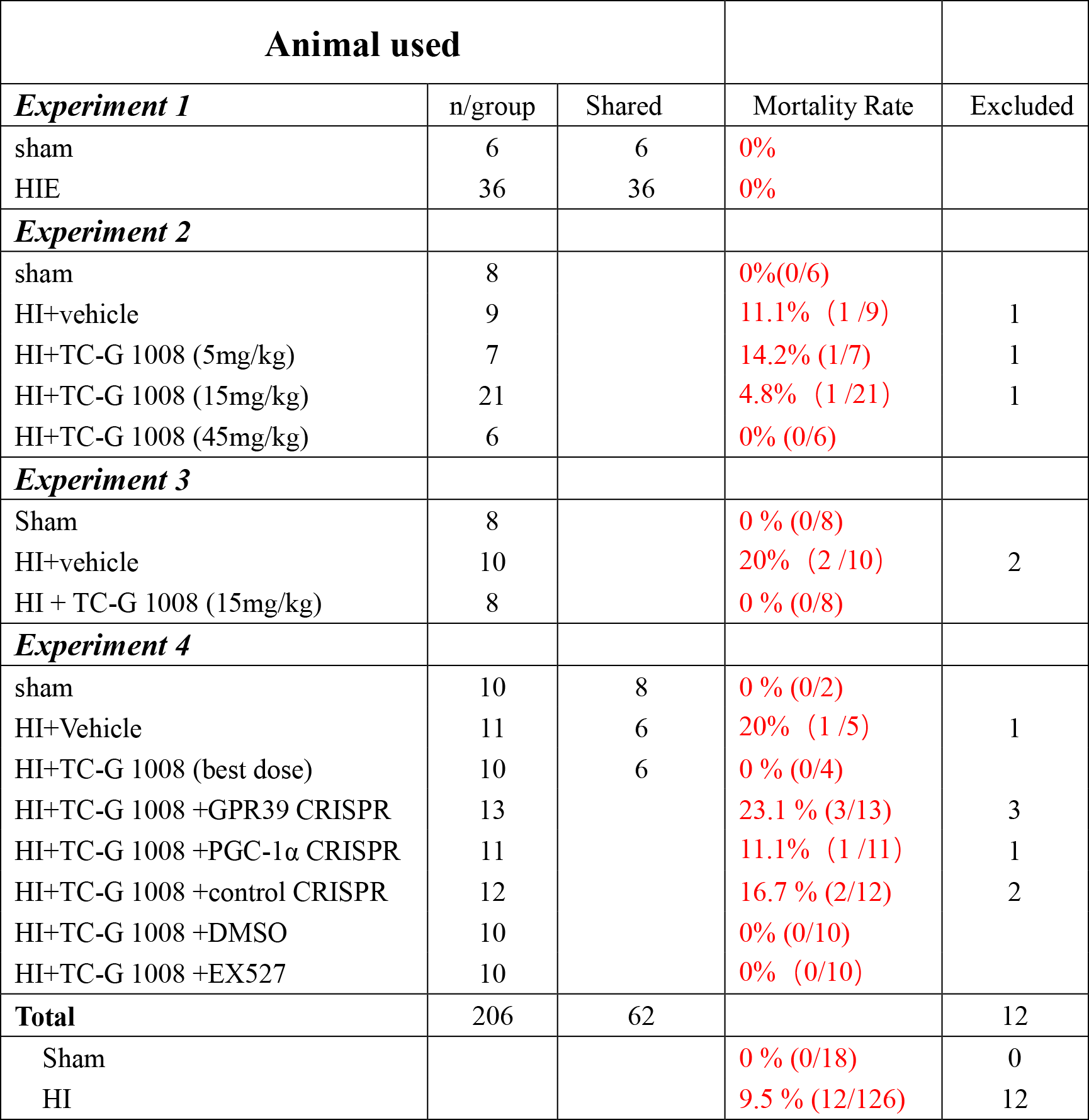

Supplement: Supplementary file 1 — Additional file 1: Details of animals used in this study. [file 12974_2021_2289_MOESM1_ESM.png]
